# Impact of testicular & other genito-urinary cancers on Egypt vesion2030/SDGs

**DOI:** 10.1080/2090598X.2023.2239111

**Published:** 2023-08-09

**Authors:** Abdel-Aziz A. Elgamal

**Affiliations:** Urology, Uro-Oncology, NIUN, Chairman, Egypt Urological Guidelines, MOHP, Head, Prostate Clinic, Cairo, Egypt

I read with interest the manuscript titled “Clinical Characteristics and treatment outcomes of Germ Cell Tumor in Jordan: a Tertiary Center Experience” in this issue of the Arab Journal of Urology [1] Whereas, providing healthy well being and improving survival, evenfor sick patients, is a top priority of the UN-Sustainable Development Goals (SDGs) 2030. Obviously, that also applies to cancer patients world-wide. In 2020, a large Arab country like Egypt (100 Million population), it has estimated total number of 134,632cancer patients (by incidence: I) & that of 89,042 deaths (by mortality: M), with M/I-Ratio of 66.1%, causing the worst impact on SDGs than that of COVID-19 [2]. That is alhough ancient medicine dated +5000 years & modern medical school established 1840), i.e. as compared to the leading American medical school in the new England area.

In contrast, testicular cancer (TC) exhibited the following numbers: I: 273, M: 48, and an M/I-ratio of 17.6%. This M/I-ratio is significantly superior to that of all other genitourinary cancers (GUCa). When compared to the M/I-ratios of other GUCa, such as bladder cancer (I: 10655, M: 6,170, M/I-ratio: 57%), prostate cancer (I: 4,767, M: 2,227, M/I-ratio: 46.7%), and kidney cancer (I: 2,208, M: 1,105, M/I-ratio: 50%), the M/I-ratio for TC stands out as the most favorable reflection of survival [3].

ratio of 17.6% as an indication of enhanced survival in TC compared to other cancers and GUCa in Egypt and the Arab World, it is noteworthy to draw attention to President Biden’s recent revival of the ‘Cancer Moonshot.’ This initiative aims to achieve new objectives, including a 50% reduction in cancer mortality over a 25-year period and an improvement in the well-being of individuals affected by cancer and cancer survivors. Simultaneously, the cancer community has made significant strides towards three ambitious goals: expediting scientific discoveries in cancer research, fostering collaboration among various stakeholders, and enhancing the exchange of cancer-related data. These efforts primarily concentrate on areas of cancer research that are most likely to yield beneficial outcomes due to increased investment. The Cancer Moonshot initiative has effectively united a large community consisting of patients, advocates, researchers, and clinicians, all dedicated to advancing research with the ultimate goal of eradicating cancer as we currently understand it [4].
